# Composite endpoints in COPD: clinically important deterioration in the UPLIFT trial

**DOI:** 10.1186/s12931-020-01431-y

**Published:** 2020-07-09

**Authors:** Klaus F. Rabe, David M. G. Halpin, MeiLan K. Han, Marc Miravitlles, Dave Singh, Lars Grönke, Florian Voß, Fernando J. Martinez

**Affiliations:** 1 Member of the German Center for Lung Research (DZL), LungClinic Grosshansdorf, Wöhrendamm 80, 22927 Grosshansdorf, Germany; 2 Member of the German Center for Lung Research (DZL), Christian Albrechts University Kiel, Kiel, Germany; 3 University of Exeter Medical School, College of Medicine and Health, University of Exeter, Exeter, UK; 4 Division of Pulmonary and Critical Care, University of Michigan Health System, Ann Arbor, MI USA; 5 Pneumology Department, Hospital Universitari Vall d’Hebron, Vall d’Hebron Institut de Recerca (VHIR), Vall d’Hebron Barcelona Hospital Campus, Ciber de Enfermedades Respiratorias (CIBERES), Barcelona, Spain; 6 Medicines Evaluation Unit (MEU), University of Manchester, Manchester University NHS Foundation Trust, Manchester, UK; 7 Clinical Development, CSL Behring GmbH, Marburg, Germany; 8 Boehringer Ingelheim Pharma GmbH & Co. KG, Ingelheim am Rhein, Germany; 9 Department of Internal Medicine, Weill Cornell School of Medicine, New York, NY USA

**Keywords:** Tiotropium, Lung function, Exacerbations

## Abstract

**Background:**

Assessments of lung function, exacerbations and health status are common measures of chronic obstructive pulmonary disease (COPD) progression and treatment response in clinical trials. We hypothesised that a composite endpoint could more holistically assess clinically important deterioration (CID) in a COPD clinical trial setting.

**Methods:**

A composite endpoint was tested in a post hoc analysis of 5652 patients with Global Initiative for Chronic Obstructive Lung Disease (GOLD) 2–4 COPD from the 4-year UPLIFT study. Patients received tiotropium 18 μg or placebo.

**Results:**

The composite endpoint included time to first confirmed decrease in trough forced expiratory volume in 1 s (FEV_1_) ≥100 mL, confirmed increase in St. George’s Respiratory Questionnaire (SGRQ) total score ≥ 4 units, or moderate/severe exacerbation. Most patients (> 80%) experienced CID, with similar incidence among GOLD subgroups. Most confirmed trough FEV_1_ (74.6–81.6%) and SGRQ (72.3–78.1%) deteriorations were sustained across the study and in all GOLD subgroups. Patients with CID more frequently experienced subsequent exacerbation (hazard ratio [HR] 1.79; 95% confidence interval [CI] 1.67, 1.92) or death (HR 1.21; 95% CI 1.06, 1.39) by Month 6. CID was responsive to bronchodilator treatment.

**Conclusions:**

Composite endpoints provide additional information on COPD progression and treatment effects in clinical trials.

**Trial registration:**

ClinicalTrials.gov NCT00144339.

**Graphical abstract:**

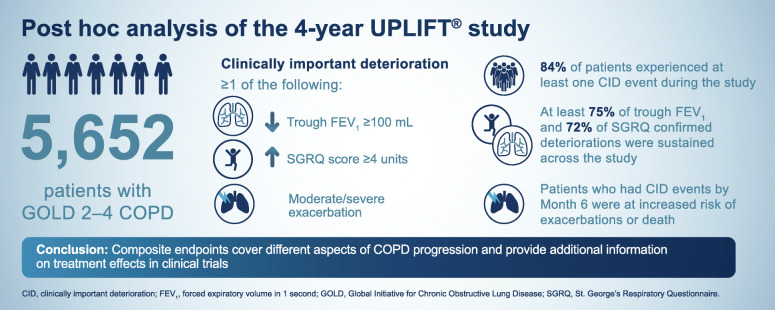

## Background

Chronic obstructive pulmonary disease (COPD) is a chronic, progressive, heterogeneous disease. The manifestation of disease progression varies over time and between patients. Despite this, most clinical trials conducted in COPD typically focus on a single primary outcome such as forced expiratory volume in 1 s (FEV_1_), exacerbation frequency or, less frequently, mortality.

The impact of interventions on disease progression has been measured by the annual rate of decline in FEV_1_ over several years [1]. However, there are limitations to this approach. Individuals with a slower rate of decline may dilute any observable treatment benefit in rapidly progressing subgroups. Moreover, individuals with a rapid decline may discontinue studies early, underestimating the true mean rate of decline in the control arm [2].

Primary analyses of clinical trials typically report group mean results, which can be insufficient to detect clinically important changes at the individual patient level. Furthermore, the focus on only one dimension of COPD may misrepresent real improvements that are meaningful to patients.

Measuring clinically important deterioration (CID) in terms of the most impactful events at the individual patient level might provide a significant benefit in studying the progression and effects of COPD in clinical trials. The three events included in this composite endpoint – trough FEV_1_, St. George’s Respiratory Questionnaire (SGRQ) score and moderate/severe exacerbation – have been previously used by Singh et al. [3], Anzueto et al. [4] and Greulich et al. [5], and were selected because they are commonly used in clinical trials and are known to have an impact on patients with COPD.

To explore the composite endpoint of CID further, we used a post hoc analysis of the 4-year UPLIFT study. The objectives of this analysis were to test the validity of CID when only including FEV_1_ and SGRQ events that were confirmed at a subsequent visit, to prove that CID predicts future outcomes, and to explore other elements of CID.

## Methods

This post hoc analysis assessed time to first CID as time to the first occurrence of at least one of the following: decrease in trough FEV_1_ from baseline ≥100 mL, increase in SGRQ total score from baseline ≥4 units or moderate/severe exacerbation (the same components as suggested by Singh et al. [3]). Changes in FEV_1_ and SGRQ score were always calculated from baseline. Changes in FEV_1_ were assessed using pre-bronchodilation values, in line with previous studies assessing CID [6–8] and reflecting real-world clinical practice for FEV_1_ monitoring. A decrease in trough FEV_1_ ≥ 100 mL is considered to be the minimum clinically important change perceived by patients [9, 10] and is within the defined range suggested by the American Thoracic Society/European Respiratory Society task force [11], whereas an increase in SGRQ total score ≥ 4 units is considered the minimum clinically important change in quality of life [12].

Unlike for the composite endpoint published by Singh et al. [3], we only included confirmed FEV_1_ and SGRQ deteriorations, i.e. events that were present during at least two consecutive assessments (5 or 6 months apart). This excluded short-term fluctuations in the disease, which could provide an unreliable indication of CID. If no further assessment was available, but the patient discontinued study medication or died, the event was also considered as confirmed. Confirmed events were not required for exacerbations of COPD.

We have used the term “sustained” to refer to deteriorations that were then maintained at almost every subsequent visit.

### Study design

Study design details have been previously reported [13] and are briefly summarised below. UPLIFT (ClinicalTrials.gov: NCT00144339) was a 4-year, randomised, double-blind, parallel-group study comparing tiotropium 18 μg, administered once daily via the HandiHaler®, with matching placebo [14]. The UPLIFT study was conducted in 37 countries [14]. Patients were aged ≥40 years, with a smoking history of ≥10 pack-years and moderate-to-very severe COPD (Global Initiative for Chronic Obstructive Lung Disease [GOLD] 2–4 [15]). For further details, see the Supplementary Methods. The protocol was approved by the ethics committee at each centre, and all patients provided written, informed consent.

Spirometric testing was performed at randomisation, at the Day 30 visit and at visits every 6 months up to Month 48. SGRQ was assessed at randomisation and every 6 months up to Month 48. Exacerbations and associated hospital admissions were recorded on case report forms at every visit. The two primary endpoints were pre- and post-bronchodilation yearly rate of decline in mean FEV_1_.

#### Statistical analysis

For time-to-event endpoints, hazard ratios (HRs), 95% confidence intervals (CIs) and *P* values were calculated using a Cox proportional hazards model. Patients without CID events were censored at the treatment stop date.

To assess the association of CID with future outcomes, patients experiencing a CID event within the first 6 months were compared with those not experiencing the event. For this analysis, the time to first moderate/severe exacerbation was calculated from Month 6 (180 days) to the first subsequent event or treatment discontinuation. Time to death was calculated from Month 6 (180 days) to the date of death or the end of the vital status follow-up (Day 1470).

## Results

Patient dispositions have been reported previously [14]. Overall, 5652 patients received treatment (2811 tiotropium; 2841 placebo) and had baseline measurements for both FEV_1_ and SGRQ. GOLD stage at baseline (based on post-bronchodilator FEV_1_) was available for 5589 patients (GOLD 2: 1293 placebo, 1310 tiotropium; GOLD 3: 1266 placebo, 1239 tiotropium; GOLD 4: 250 placebo, 231 tiotropium).

### Incidence of CID

Most patients in the total population (83.9%) experienced at least one CID during the study (Table 1). Exacerbations were more frequent than FEV_1_ or SGRQ decline (Table 1).

**Table 1:** Incidence of CID and risk of first CID occurrence in total population and by GOLD stage

	Overall population	GOLD 2	GOLD 3	GOLD 4
**Total UPLIFT population (placebo and tiotropium combined)**				
Number of patients, n (%)	5652 (100.0)	2603 (100.0)	2505 (100.0)	481 (100.0)
At least one of SGRQ deterioration (decrease of ≥4 units), trough FEV_1_ decline ≥100 mL or moderate/severe exacerbation	4741 (83.9)	2175 (83.6)	2127 (84.9)	388 (80.7)
Moderate/severe exacerbation	3814 (67.5)	1615 (62.0)	1796 (71.7)	357 (74.2)
Trough FEV_1_ decline ≥100 mL	2503 (44.3)	1344 (51.6)	1031 (41.2)	100 (20.8)
SGRQ score increase ≥4 units	2339 (41.4)	1081 (41.5)	1054 (42.1)	178 (37.0)

*CID* clinically important deterioration, *FEV*_*1*_ forced expiratory volume in 1 s, *GOLD* Global Initiative for Chronic Obstructive Lung Disease, *SGRQ* St. George’s Respiratory Questionnaire

The contribution of exacerbations to the composite endpoint became more pronounced whereas the contribution of FEV_1_ became less pronounced as COPD severity (GOLD stage) increased in the total population (Table 1).

Time to first event for each component is shown in e-Figure 1.

**Fig. 1:**
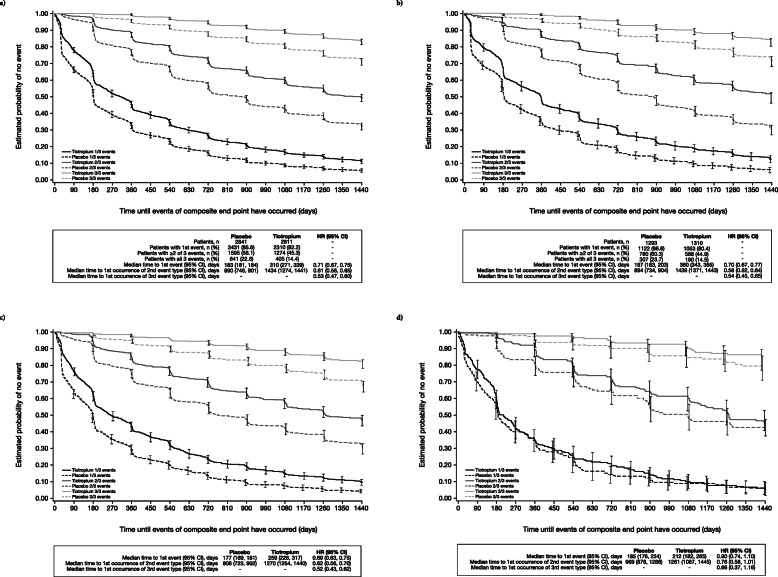
Kaplan–Meier estimates for the time to CID in the overall population and GOLD subgroups. Kaplan–Meier estimates of time to first event, two of three events, or three of three events of the components of the composite endpoint (trough FEV_1_ decline ≥100 mL, SGRQ score deterioration ≥4 units, or moderate/severe exacerbation) in (**a**) the overall population, (**b**) GOLD 2 patients, (**c**) GOLD 3 patients, and (**d**) GOLD 4 patients. - indicates either that this value could not be assessed (median was not estimable) or is not applicable (HR only displayed for time to event analysis). CI: confidence interval; FEV_1_: forced expiratory volume in 1 s; GOLD: Global Initiative for Chronic Obstructive Lung Disease; HR: hazard ratio; SGRQ: St. George’s Respiratory Questionnaire

Overall, about half of patients experienced at least two of the three events qualifying as CID, whereas fewer patients experienced all three events (Fig. 1a). A similar proportion of patients in each GOLD group experienced at least two CID events (Fig. 1b–d). The incidence of all three CID events was also similar for GOLD 2 and 3 patients, whereas few GOLD 4 patients experienced all three CID events (Fig. 1b–d).

Overall, most confirmed events were sustained at subsequent visits. Confirmed trough FEV_1_ decline was sustained at 12–48 months after the initial event in 74.6–81.6% of patients (Table 2). Confirmed SGRQ deterioration was also sustained at 12–42 months after the initial event in 72.3–78.1% of patients (Table 2). This pattern was comparable with the GOLD subgroups (e-Table 1), although patient numbers were low for the GOLD 4 subgroup.

**Table 2:** Patients with FEV_1_ decline or SGRQ deterioration in the total population

	**Patients with confirmed FEV**_**1**_**decline or SGRQ deterioration at time points after the initial decline (available assessments)**							
	**Months after confirmed FEV**_**1**_**decline or SGRQ increase**							
	6	12	18	24	30	36	42	48
**FEV**_**1**_**decline**								
No. of patients with decline, n (%)	1924/1924 (100)	1239/1661 (74.6)	1091/1447 (75.4)	895/1199 (74.6)	754/966 (78.1)	604/755 (80.0)	419/549 (76.3)	280/343 (81.6)
Mean FEV_1_ change from baseline, mL (SD)	−223 (114)	−193 (176)	−202 (187)	−214 (195)	−237 (212)	−256 (224)	−258 (230)	−280 (224)
Median FEV_1_ change from baseline mL (min, max)	−190 (−1090, −40)	−180 (−1240, 850)	−200 (−1000, 0.630)	−210 (−1070, 0.740)	−240 (−1190, 650)	−240 (−1200, 490)	−250 (−980, 460)	−270 (−920, 670)
**SGRQ deterioration**								
No. of patients with deterioration, n (%)	1785/1785 (100)	1140/1510 (75.5)	941/1301 (72.3)	776/1067 (72.7)	629/843 (74.6)	462/617 (74.9)	303/388 (78.1)	
Mean SGRQ change from baseline (SD)	12.5 (7.5)	10.6 (10.7)	10.2 (11.4)	10.7 (12.3)	11.3 (12.6)	11.8 (12.8)	12.3 (12.8)	
Median SGRQ score change from baseline (min, max)	10.5 (4.0, 53.1)	9.5 (−48.4, 53.9)	9.3 (−44.2, 67.8)	10.2 (−44.5, 69.6)	10.7 (−36.6, 54.6)	10.9 (−33.8, 50.1)	11.9 (−29.8, 47.3)	

For patients with confirmed decline at Month 1, their assessments at Months 6, 12, 18, 24, 30, 36, 42 and 48 are used as time from first confirmed deterioration. Unscheduled visits were excluded for this analysis. Only patients with confirmed FEV_1_ decline or confirmed SGRQ deterioration and available assessments at each time point after the deterioration were included in the analysis

*FEV*_*1*_ forced expiratory volume in 1 s, *SD* standard deviation, *SGRQ* St. George’s Respiratory Questionnaire

For unconfirmed events (reported at one timepoint), the proportion of patients whose FEV_1_ decline or SGRQ deterioration was sustained was lower: 51.6–71.9% of patients still had the FEV_1_ decline 6–48 months after first decline, and 52.5–65.5% still had SGRQ deterioration (e-Table 2).

In addition, in patients who had confirmed events, mean FEV_1_ remained at least 193 mL worse than baseline for the rest of the trial (Table 2). For unconfirmed events, mean FEV_1_ in patients with an event ranged from 95 mL worse than baseline at Month 6 to 142 mL worse than baseline at Month 24 and 213 mL at Month 48. In patients with SGRQ deterioration, mean increase was > 10 units for the rest of the trial for confirmed events, but ranged from 4.7 to 8.3 units for unconfirmed events.

### Relative timing of events

**Fig. 2:**
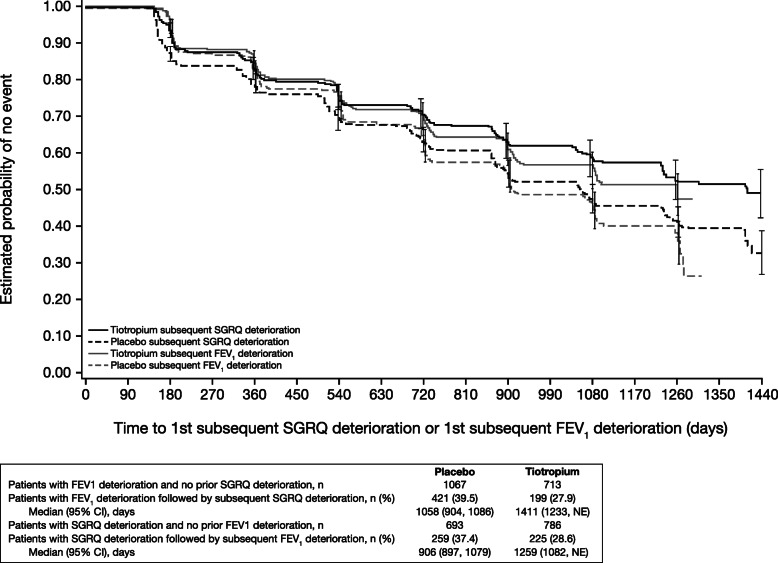
Kaplan–Meier estimates of time to first subsequent SGRQ deterioration or first subsequent FEV_1_ deterioration. Kaplan–Meier estimates of median time from FEV_1_ decline ≥100 mL to SGRQ score deterioration ≥4 units, and median time from SGRQ score deterioration ≥4 units to FEV_1_ decline ≥100 mL in the overall population. CI: confidence interval; FEV_1_: forced expiratory volume in 1 s; NE: not evaluable; SGRQ: St. George’s Respiratory Questionnaire

The pattern and timing of clinically relevant events was highly variable for individual patients. Of patients who experienced both confirmed FEV_1_ decline and SGRQ deterioration, it was unusual to experience both events at the same assessment (Table 3). The time from FEV_1_ decline to subsequent SGRQ deterioration was slightly longer than the time from SGRQ deterioration to subsequent FEV_1_ decline (Fig. 2).

**Table 3:** Timing of FEV_1_ decline and SGRQ deterioration in the overall population and GOLD subgroups

	Overall	GOLD 2	GOLD 3	GOLD 4
Patients with both confirmed FEV_1_ decline and confirmed SGRQ deterioration, n (%)	1344 (100.0)	698 (100.0)	575 (100.0)	54 (100.0)
On same assessment	240 (17.9)	121 (17.3)	108 (18.8)	9 (16.7)
FEV_1_ decline before SGRQ deterioration	620 (46.1)	350 (50.1)	239 (41.6)	20 (37.0)
SGRQ deterioration before FEV_1_ decline	484 (36.0)	227 (32.5)	228 (39.7)	25 (46.3)
Moderate/severe exacerbation before FEV_1_ and SGRQ deterioration	546 (40.6)	246 (35.2)	269 (46.8)	24 (44.4)
Patients with confirmed FEV_1_ decline and no confirmed SGRQ deterioration, n (%)	1160 (100.0)	644 (100.0)	459 (100.0)	46 (100.0)
Moderate/severe exacerbation before FEV_1_ decline	460 (39.7)	231 (35.9)	199 (43.4)	24 (52.2)
Unconfirmed SGRQ deterioration	302 (26.0)	180 (28.0)	109 (23.7)	10 (21.7)
On same assessment as confirmed FEV_1_ decline	34 (2.9)	20 (3.1)	12 (2.6)	1 (2.2)
Before confirmed FEV_1_ decline	116 (10.0)	72 (11.2)	36 (7.8)	7 (15.2)
Patients with confirmed SGRQ and no confirmed FEV_1_ deterioration, n (%)	995 (100.0)	383 (100.0)	479 (100.0)	124 (100.0)
Moderate/severe exacerbation before SGRQ deterioration	574 (57.7)	184 (48.0)	305 (63.7)	80 (64.5)
Unconfirmed FEV_1_ deterioration	284 (28.5)	141 (36.8)	126 (26.3)	15 (12.1)
On same assessment as confirmed SGRQ deterioration	50 (5.0)	23 (6.0)	24 (5.0)	3 (2.4)
Before confirmed SGRQ deterioration	151 (15.2)	80 (20.9)	64 (13.4)	5 (4.0)

Unscheduled visits were excluded for this analysis

*FEV*_*1*_ forced expiratory volume in 1 s, *GOLD* Global Initiative for Chronic Obstructive Lung Disease, *SGRQ* St. George’s Respiratory Questionnaire

For patients who experienced both FEV_1_ decline and SGRQ deterioration, those with less spirometric obstruction appeared more likely to experience confirmed FEV_1_ decline prior to confirmed SGRQ deterioration (GOLD 2: 50.1%; GOLD 3: 41.6%; GOLD 4: 37.0%) (Table 3).

Exacerbations demonstrated a greater contribution to the composite endpoint in more severe patients. Patients with more severe COPD were more likely to experience an exacerbation prior to experiencing FEV_1_ decline or SGRQ deterioration (Table 3).

### Response to treatment

The time to first CID event, and time to first occurrence of the individual components, was sensitive to therapeutic intervention (Table 4). Time to first CID, two CID events and all three CID events was longer with tiotropium than with placebo (Table 4 and Fig. 1a). This trend was observed in GOLD 2 and 3 subgroups, but less so with GOLD 4 patients (Fig. 1b–d).

**Table 4:** Treatment comparison of time to first CID in the overall population and by GOLD stage

	**Event, n (%)**		**Time to first event treatment comparison (tiotropium–placebo)**	
	**Tiotropium 18 μg**	**Placebo**	**HR (95% CI)**	***P*value**
Overall	2811 (100.0)	2841 (100.0)		
SGRQ deterioration, trough FEV_1_ decline ≥100 mL, moderate/severe exacerbation	2310 (82.2)	2431 (85.6)	0.71 (0.67, 0.75)	< 0.0001
Moderate/severe exacerbation	1884 (67.0)	1930 (67.9)	0.86 (0.81, 0.92)	< 0.0001
Trough FEV_1_ decline ≥100 mL	1028 (36.6)	1475 (51.9)	0.53 (0.49, 0.58)	< 0.0001
SGRQ score increase ≥4 units	1077 (38.3)	1262 (44.4)	0.72 (0.66, 0.78)	< 0.0001
GOLD 2	1310 (100.0)	1293 (100.0)		
SGRQ deterioration, trough FEV_1_ decline ≥100 mL, moderate/severe exacerbation	1053 (80.4)	1122 (86.8)	0.70 (0.65, 0.77)	< 0.0001
Moderate/severe exacerbation	780 (59.5)	835 (64.6)	0.83 (0.75, 0.92)	0.0002
Trough FEV_1_ decline ≥100 mL	564 (43.1)	780 (60.3)	0.54 (0.49, 0.61)	< 0.0001
SGRQ score increase ≥4 units	487 (37.2)	594 (45.9)	0.71 (0.63, 0.80)	< 0.0001
GOLD 3	1239 (100.0)	1266 (100.0)		
SGRQ deterioration, trough FEV_1_ decline ≥100 mL, moderate/severe exacerbation	1036 (83.6)	1091 (86.2)	0.69 (0.63, 0.75)	< 0.0001
Moderate/severe exacerbation	896 (72.3)	900 (71.1)	0.86 (0.78, 0.94)	0.0010
Trough FEV_1_ decline ≥100 mL	413 (33.3)	618 (48.8)	0.51 (0.45, 0.58)	< 0.0001
SGRQ score increase ≥4 units	491 (39.6)	563 (44.5)	0.71 (0.63, 0.81)	< 0.0001
GOLD 4	231 (100.0)	250 (100.0)		
SGRQ deterioration, trough FEV_1_ decline ≥100 mL, moderate/severe exacerbation	197 (85.3)	191 (76.4)	0.90 (0.74, 1.10)	0.3079
Moderate/severe exacerbation	186 (80.5)	171 (68.4)	1.00 (0.81, 1.23)	0.9798
Trough FEV_1_ decline ≥100 mL	41 (17.7)	59 (23.6)	0.57 (0.39, 0.86)	0.0066
SGRQ score increase ≥4 units	90 (39.0)	88 (35.2)	0.83 (0.62, 1.11)	0.2105

*CI* confidence interval, *CID* clinically important deterioration, *FEV*_*1*_ forced expiratory volume in 1 s, *GOLD* Global Initiative for Chronic Obstructive Lung Disease, *HR* hazard ratio, *SGRQ* St. George’s Respiratory Questionnaire

### Risk of future exacerbations and death

Patients who had CID events by Month 6 were more likely to experience a moderate or severe exacerbation (HR 1.79; 95% CI 1.67, 1.92), a severe exacerbation (HR 1.67; 95% CI 1.49, 1.86) or death (HR 1.21; 95% CI 1.06, 1.39) (Table 5). The increase in the risk of exacerbations was qualitatively similar for GOLD 2–4 subgroups (Table 5).

**Table 5:** Risk of exacerbation or death from Month 6 onwards by CID status at Month 6 in the overall population and by GOLD

Outcome at Month 6	Patients with any CID event vs. patients without, HR (95% CI)	Patients with confirmed FEV_1_ decline vs. patients without, HR (95% CI)	Patients with confirmed SGRQ deterioration vs. patients without, HR (95% CI)	Patients with moderate/severe exacerbation vs. patients without, HR (95% CI)
Overall population				
Moderate/severe exacerbation	1.79 (1.67, 1.92)	1.11 (1.02, 1.22)	1.30 (1.18, 1.43)	2.36 (2.20, 2.53)
Severe exacerbation	1.67 (1.49, 1.86)	1.06 (0.92, 1.23)	1.66 (1.44, 1.91)	1.88 (1.68, 2.11)
Death up to Day 1470	1.21 (1.06, 1.39)	1.09 (0.92, 1.31)	1.27 (1.05, 1.54)	1.22 (1.05, 1.41)
GOLD 2				
Moderate/severe exacerbation	1.73 (1.56, 1.92)	1.15 (1.01, 1.30)	1.24 (1.07, 1.44)	2.49 (2.23, 2.78)
Severe exacerbation	1.58 (1.30, 1.92)	1.23 (0.97, 1.55)	1.77 (1.37, 2.27)	1.79 (1.46, 2.21)
Death up to Day 1470	1.21 (0.95, 1.55)	1.26 (0.94, 1.68)	1.36 (0.98, 1.90)	1.01 (0.75, 1.35)
GOLD 3				
Moderate/severe exacerbation	1.84 (1.67, 2.04)	1.18 (1.03, 1.35)	1.32 (1.15, 1.52)	2.21 (1.99, 2.45)
Severe exacerbation	1.69 (1.45, 1.96)	1.08 (0.88, 1.33)	1.57 (1.29, 1.92)	1.79 (1.54, 2.09)
Death up to Day 1470	1.20 (0.99, 1.45)	1.17 (0.91, 1.51)	1.30 (1.00, 1.70)	1.15 (0.94, 1.41)
GOLD 4				
Moderate/severe exacerbation	1.84 (1.47, 2.32)	1.35 (0.85, 2.15)	1.57 (1.14, 2.16)	1.97 (1.55, 2.50)
Severe exacerbation	1.69 (1.26, 2.25)	2.38 (1.46, 3.88)	1.81 (1.23, 2.67)	1.46 (1.09, 1.97)
Death up to Day 1470	1.16 (0.84, 1.60)	1.52 (0.88, 2.63)	1.00 (0.60, 1.66)	1.22 (0.87, 1.70)

Time to death was calculated from Month 6 (180 days) to the date of death or the end of the vital status follow-up (Day 1470)

*CI* confidence interval, *CID* clinically important deterioration, *GOLD* Global Initiative for Chronic Obstructive Lung Disease, *HR* hazard ratio, *SGRQ* St. George’s Respiratory Questionnaire

When the composite endpoint was broken down into its component events, the HRs for future exacerbations were smaller for FEV_1_ decline and SGRQ deterioration by Month 6 than for the composite endpoint in the overall population, and among GOLD 2 and GOLD 3 COPD patients (Table 5). Exacerbations within 6 months had higher HRs for any exacerbation and for severe exacerbations than the composite endpoint.

For unconfirmed events, the HRs for long-term outcomes were lower than for the sustained events (Table 5 and e-Table 3).

Investigating future events by CID status at Month 12 showed similar results (e-Table 4).

### Mortality analysis with CID

Additional analyses using time to composite event or time to one of the component events as a time-varying covariate were performed. The HR for death for patients with a CID event versus patients without an event was 1.69 (95% CI 1.42, 2.01) (e-Table 5).

Using a stepwise Cox regression model to adjust for important baseline predictors of mortality had little effect on the predictive performance of the composite (e-Table 5). When all three components were included as separate predictors, all were associated with increased mortality risk (e-Table 5).

To validate these findings, the results in e-Tables 4, 6 and 7 are presented for the placebo and tiotropium arms separately. The HRs are slightly higher in the tiotropium arm, which may be related to the larger number of events in the placebo arm before Month 6. The results in e-Tables 6 and 7 are similar between arms and confirm the results in the total population.

## Discussion

Composite endpoints have only recently been introduced in post hoc analyses of COPD clinical trials [3–6, 16]. Here, we conducted a post hoc analysis of the UPLIFT study. This analysis demonstrated the importance of using confirmed events in CID analysis and that CID predicts future outcomes. It also confirmed that the components of this composite endpoint behaved differently based on the baseline FEV_1_ of the individual patient. These data suggest that sustained decline in trough FEV_1_, sustained deterioration in SGRQ score of ≥4 units and a moderate/severe exacerbation are appropriate components of a composite endpoint for the assessment of CID in patients enrolled in COPD clinical trials. Earlier analyses of the UPLIFT trial have focused on exacerbations or a composite endpoint of the more severe events (exacerbations, respiratory failure, death and trial withdrawal due to worsening COPD), which do not provide an in-depth view of the impact of COPD on patient symptoms or quality of life [16, 17]. In the current analysis we focus on a composite endpoint of validated clinically important criteria (FEV_1_, SGRQ and exacerbations) to provide a more complete assessment of the impact on patients.

The individual components of the composite endpoint comprise characteristics of COPD that impact patient well-being, are clinically relevant events for the patient and predict future outcomes [15]. Although there are other parameters that could be included in such an endpoint, the components included are relatively easy to include in clinical trials and have established minimum clinically important differences.

Most deteriorations in FEV_1_ and SGRQ that were confirmed at a second visit were maintained for the rest of the 4-year UPLIFT study. Some publications of composite endpoints in COPD have not required confirmation at a subsequent visit [3, 16]. We believe that counting only confirmed FEV_1_ and SGRQ deteriorations improves the reliability of the composite endpoint, as it excludes short-term variation and inconsistent measurements. This is supported by the low proportion of patients with unconfirmed events whose FEV_1_ or SGRQ deterioration is sustained at subsequent timepoints, and by the lower HRs for long-term outcomes with unconfirmed events compared with confirmed events.

Our analysis demonstrated that the components of the composite endpoint rarely occur at the same time in an individual patient. Most patients experience decline of trough FEV_1_, deterioration of SGRQ score and moderate/severe exacerbations on an individualised time scale. This supports the value of individual components in a composite endpoint. The stepwise regression data also show that each component independently contributes to increased mortality risk. The composite endpoint is also sensitive to pharmacological treatment, and is similar to the findings of Singh et al., who observed a reduction in first CID with umeclidinium/vilanterol versus placebo in a post hoc study of the same composite endpoint [3]. Other post hoc analyses have used slightly different composite endpoints: FEV_1_, SGRQ and Transition Dyspnea Index focal score [6]; FEV_1_ or Transition Dyspnea Index; an increase in SGRQ; and a moderate-to-severe COPD exacerbation [4].

In all the publications that included FEV_1_, the strongest driver of CID in each of the analysis populations was lung function [3–6]. In contrast to these previous results, the most commonly reported endpoint in our study was exacerbations, perhaps because the UPLIFT study was 4 years long compared with the shorter (maximum 26 weeks) duration of the previous studies [3]. Our analysis showed a high overall frequency of CID for both treatment arms, which is expected due to the long study duration.

Lastly, we have shown that patients considered to have a CID early in the UPLIFT study (within the first 6 months) had worse outcomes for the 42-month remainder of the study; this was also confirmed in an analysis using CID as a time-varying covariate. These outcomes support results from previous analyses of the shorter TORCH and ECLIPSE studies. The 4-year length of our study provided valuable information on sustained CID and the relationship between clinically important events that could not be ascertained in clinical trials of shorter duration.

The study had limitations. In addition, relatively few patients with GOLD 4 lung function impairment were enrolled. Additionally, GOLD 4 patients have a lower baseline FEV_1_ than GOLD 2 or 3 patients, and as such, declines in FEV_1_ of ≥100 mL were less common, and would be expected to be more debilitating, in these patients. This should be considered in future studies, where percentage declines may be considered as an alternative clinically significant decline. The composite index considers the parameters SGRQ and moderate/severe exacerbations, which could be seen as subjective; therefore, it is possible that this could introduce some variability in the results. Also, this was a post hoc analysis, although the large population and long follow-up time allowed for a satisfactory number of events to be observed.

## Conclusions

We believe these results indicate that a composite endpoint of CID is a promising endpoint to assess disease activity in COPD clinical trials and may be a useful outcome that helps clinicians interpret the implications of trial results for individual patient management. Development of prospective studies is required to determine whether patients who experience disease progression (i.e. those who experience CID) at an increased rate can be identified earlier. By stratifying patients based on time to CID in a clinical trial database, it may be possible to identify characteristics that are associated with longer-term poor outcomes that could be useful for identifying which patients require further treatment earlier. Moreover, the composite endpoint may also serve to reduce patient numbers in clinical trials, as large numbers of patients are required to generate enough statistical power to detect a single outcome within patients with moderate COPD [18]. The length of trials may also be reduced, thereby limiting challenges such as patient discontinuation and cost that are prohibitive in trials of increased duration. Prospective studies are needed on the use of this concept to understand the sensitivity and efficacy of current and potential therapies.

## Supplementary information

**Additional file 1: Supplementary Table 1.** Patients with FEV_1_ decline or SGRQ increase 6, 12, 18, 24, 30, 36, 42, and 48 months after the first confirmed FEV_1_ decline or SGRQ increase by GOLD 2, GOLD 3, and GOLD 4. **Supplementary Table 2.** Patients with FEV_1_ decline or SGRQ deterioration in the total UPLIFT population 6, 12, 18, 24, 30, 36, 42, and 48 months after the initial unconfirmed FEV_1_ decline or SGRQ deterioration. **Supplementary Table 3.** Risk of exacerbation or death by unconfirmed clinically important deterioration status at Months 6 and 12. **Supplementary Table 4.** Risk of exacerbation or death by confirmed clinically important deterioration status at Month 6 in the tiotropium and placebo arms, and at Month 12 in the tiotropium and placebo arms and total population. **Supplementary Table 5.** Risk of exacerbation or death by confirmed clinically important deterioration status calculated using clinically important deterioration event as a time-varying covariate. **Supplementary Table 6.** Patients with FEV_1_ decline or SGRQ deterioration in the total UPLIFT population 6, 12, 18, 24, 30, 36, and 42 months after the initial confirmed FEV_1_ decline or SGRQ deterioration: Tiotropium and placebo. **Supplementary Table 7.** Timing of FEV_1_ decline and SGRQ deterioration relative to each other in the tiotropium and placebo arms. **Supplementary Figure 1.** Time to first event for (A) trough FEV_1_ decline ≥100 mL, (B) SGRQ increase ≥4 units and (C) moderate/severe exacerbation.

## Data Availability

Data are available from the corresponding author upon reasonable request.

## Abbreviations

CI: Confidence interval
CID: Clinically important deterioration
COPD: Chronic obstructive pulmonary disease
FEV_1_: Forced expiratory volume in 1 s
GOLD: Global Initiative for Chronic Obstructive Lung Disease
HR: Hazard ratio
SGRQ: St. George’s Respiratory Questionnaire
