# Supplementary material for: Composite endpoints in COPD: clinically important deterioration in the UPLIFT trial
Source: Respir Res. 2020 Jul 9;21:177. doi: 10.1186/s12931-020-01431-y (PMC7350568; doi:10.1186/s12931-020-01431-y)
Supplement: Supplementary file 1 — Additional file 1: Supplementary Table 1. Patients with FEV1 decline or SGRQ increase 6, 12, 18, 24, 30, 36, 42, and 48 months after the first confirmed FEV1 decline or SGRQ increase by GOLD 2, GOLD 3, and GOLD 4. Supplementary Table 2. Patients with FEV1 decline or SGRQ deterioration in the total UPLIFT population 6, 12, 18, 24, 30, 36, 42, and 48 months after the initial unconfirmed FEV1 decline or SGRQ deterioration. Supplementary Table 3. Risk of exacerbation or death by unconfirmed clinically important deterioration status at Months 6 and 12. Supplementary Table 4. Risk of exacerbation or death by confirmed clinically important deterioration status at Month 6 in the tiotropium and placebo arms, and at Month 12 in the tiotropium and placebo arms and total population. Supplementary Table 5. Risk of exacerbation or death by confirmed clinically important deterioration status calculated using clinically important deterioration event as a time-varying covariate. Supplementary Table 6. Patients with FEV1 decline or SGRQ deterioration in the total UPLIFT population 6, 12, 18, 24, 30, 36, and 42 months after the initial confirmed FEV1 decline or SGRQ deterioration: Tiotropium and placebo. Supplementary Table 7. Timing of FEV1 decline and SGRQ deterioration relative to each other in the tiotropium and placebo arms. Supplementary Figure 1. Time to first event for (A) trough FEV1 decline ≥100 mL, (B) SGRQ increase ≥4 units and (C) moderate/severe exacerbation. [file 12931_2020_1431_MOESM1_ESM.docx]

**Composite endpoints in COPD: clinically important deterioration in the UPLIFT trial**

Klaus F. Rabe, David Halpin, MeiLan K. Han, Marc Miravitlles, Dave Singh, Lars Grönke, Florian Voß, and Fernando Martinez

**Supplementary Material**

**Contents**

**Supplementary methods**

**Supplementary Table 1** Patients with FEV_1_ decline or SGRQ increase 6, 12, 18, 24, 30, 36, 42, and 48 months after the first confirmed FEV_1_ decline or SGRQ increase by GOLD 2, GOLD 3, and GOLD 4

**Supplementary Table 2** Patients with FEV_1_ decline or SGRQ deterioration in the total UPLIFT population 6, 12, 18, 24, 30, 36, 42, and 48 months after the initial unconfirmed FEV_1_ decline or SGRQ deterioration

**Supplementary Table 3** Risk of exacerbation or death by unconfirmed clinically important deterioration status at Months 6 and 12

**Supplementary Table 4** Risk of exacerbation or death by confirmed clinically important deterioration status at Month 6 in the tiotropium and placebo arms, and at Month 12 in the tiotropium and placebo arms and total population

**Supplementary Table 5** Risk of exacerbation or death by confirmed clinically important deterioration status calculated using clinically important deterioration event as a time-varying covariate

**Supplementary Table 6** Patients with FEV_1_ decline or SGRQ deterioration in the total UPLIFT population 6, 12, 18, 24, 30, 36, and 42 months after the initial confirmed FEV_1_ decline or SGRQ deterioration: Tiotropium and placebo

**Supplementary Table 7** Timing of FEV_1_ decline and SGRQ deterioration relative to each other in the tiotropium and placebo arms

**Supplementary Figure 1** Time to first event for (A) trough FEV_1_ decline ≥100 mL, (B) SGRQ increase ≥4 units and (C) moderate/severe exacerbation

**Supplementary methods – key inclusion and exclusion criteria**

Patients were aged ≥40 years old, with a smoking history of ≥10 pack-years and a diagnosis of moderate-to-very severe chronic obstructive pulmonary disease (COPD; Global Initiative for Chronic Obstructive Lung Disease [GOLD] 2–4), post-bronchodilator forced expiratory volume in 1 second (FEV_1_) ≤70% predicted, and an FEV_1_ ≤70% of forced vital capacity. Key exclusion criteria included a history of asthma or pulmonary resection, COPD exacerbation or respiratory infection within 4 weeks of screening, use of supplemental oxygen for ≥12 hours per day, or the presence of a coexisting illness that could preclude participation or interfere with study results.

Patients continued all previously prescribed concomitant respiratory medications other than inhaled anticholinergics, and there were no restrictions for medications prescribed for treatment of exacerbations[1].

**Supplementary Table 1** Patients with FEV_1_ decline or SGRQ increase 6, 12, 18, 24, 30, 36, 42 and 48 months after the first confirmed FEV_1_ decline or SGRQ increase by GOLD 2, GOLD 3, and GOLD 4

|  | **Months after confirmed FEV_1_ decline or SGRQ increase – GOLD 2** | | | | | | | |
| --- | --- | --- | --- | --- | --- | --- | --- | --- |
|  | **6** | **12** | **18** | **24** | **30** | **36** | **42** | **48** |
| FEV_1_ decline, n (%) | 1,081/1,081 (100.0) | 721/945 (76.3) | 640/843 (75.9) | 552/721 (76.6) | 453/582 (77.8) | 382/460 (83.0) | 253/334 (75.7) | 175/209 (83.7) |
| SGRQ decline, n (%) | 844/844 (100.0) | 527/730 (72.2) | 448/638 (70.2) | 354/521 (67.9) | 287/403 (71.2) | 214/295 (72.5) | 148/200 (74.0) |  |
|  | **Months after confirmed FEV_1_ decline or SGRQ increase – GOLD 3** | | | | | | | |
|  | **6** | **12** | **18** | **24** | **30** | **36** | **42** | **48** |
| FEV_1_ decline, n (%) | 757/757 (100.0) | 465/644 (72.2) | 406/543 (74.8) | 316/432 (73.1) | 271/348 (77.9) | 206/272 (75.7) | 153/195 (78.5) | 97/124 (78.2) |
| SGRQ decline, n (%) | 798/798 (100.0) | 534/668 (79.9) | 426/569 (74.9) | 366/474 (77.2) | 296/380 (77.9) | 219/280 (78.2) | 133/161 (82.6) |  |
|  | **Months after confirmed FEV_1_ decline or SGRQ increase – GOLD 4** | | | | | | | |
|  | **6** | **12** | **18** | **24** | **30** | **36** | **42** | **48** |
| FEV_1_ decline, n (%) | 65/65 (100.0) | 38/51 (74.5) | 29/40 (72.5) | 16/28 (57.1) | 18/21 (85.7) | 9/11 (81.8) | 7/10 (70.0) | 4/4 (100.0) |
| SGRQ decline, n (%) | 123/123 (100.0) | 68/96 (70.8) | 57/79 (72.2) | 46/60 (76.7) | 38/51 (74.5) | 22/33 (66.7) | 16/21 (76.2) |  |

FEV_1_: forced expiratory volume in 1 second; GOLD: Global Initiative for Chronic Obstructive Lung Disease; SGRQ: St. George’s Respiratory Questionnaire.
For patients with confirmed decline at Month 1, their assessments at Months 6, 12, 18, 24, 30, 36, 42, and 48 are used as time from first confirmed deterioration. Unscheduled visits were excluded for this analysis. Only patients with confirmed FEV_1_ decline or confirmed SGRQ increase and their available assessments at each time point after the deterioration were included in the analysis.

The Kaplan‒Meier plots for SGRQ score and FEV_1_ appear as stepwise because they are measured every 6 months, whereas exacerbation events could be recorded at any time.

**Supplementary Table 2** Patients with FEV_1_ decline or SGRQ deterioration in the total UPLIFT population 6, 12, 18, 24, 30, 36, 42 and 48 months after the initial unconfirmed FEV_1_ decline or SGRQ deterioration

|  | **Patients with unconfirmed FEV_1_ decline or SGRQ deterioration at time points after the initial decline (available assessments)** | | | | | | | |
| --- | --- | --- | --- | --- | --- | --- | --- | --- |
|  | **Months after first FEV_1_ decline or SGRQ increase** | | | | | | | |
|  | **6** | **12** | **18** | **24** | **30** | **36** | **42** | **48** |
| FEV_1_ decline, n (%) | 1,431/2,771 (51.6) | 1,357/2,466 (55.0) | 1,286/2,194 (58.6) | 1,164/1,913 (60.8) | 1,030/1,610 (64.0) | 875/1,307 (66.9) | 657/1,011 (65.0) | 440/612 (71.9) |
| SGRQ deterioration, n (%) | 1,370/2,611 (52.5) | 1,289/2,315 (55.7) | 1,135/2,046 (55.5) | 1,042/1,749 (59.6) | 883/1,445 (61.1) | 707/1,140 (62.0) | 468/714 (65.5) |  |

FEV_1_: forced expiratory volume in 1 second; SGRQ: St. George’s Respiratory Questionnaire.
Unscheduled visits were excluded for this analysis. Only patients with FEV_1_ decline or SGRQ deterioration and their available assessments at each time point after the deterioration were included in the analysis.

**Supplementary Table 3** Risk of exacerbation or death by unconfirmed clinically important deterioration status at Months 6 and 12

| **Outcome at Month 6** | **Patients with any CID event vs. patients without,  HR (95% CI)** | **Patients with FEV_1_ decline (unconfirmed) vs. patients without, HR (95% CI)** | **Patients with SGRQ deterioration (unconfirmed) vs. patients without, HR (95% CI)** | **Patients with moderate/severe exacerbation vs. patients without, HR (95% CI)** |
| --- | --- | --- | --- | --- |
| Moderate/severe exacerbation Severe exacerbation Death up to Day 1,470 | 1.56 (1.46, 1.68)  1.52 (1.35, 1.70)  1.14 (0.99, 1.31) | 1.07 (1.00, 1.15)  0.98 (0.87, 1.11)  1.04 (0.89, 1.20) | 1.20 (1.11, 1.29)  1.41 (1.25, 1.59)  1.03 (0.87, 1.21) | 2.36 (2.20, 2.53)  1.88 (1.68, 2.11)  1.22 (1.05, 1.41) |
| **Outcome at Month 12** | **Patients with any CID event vs. patients without,  HR (95% CI)** | **Patients with FEV_1_ decline vs. patients without, HR (95% CI)** | **Patients with SGRQ deterioration vs. patients without, HR (95% CI)** | **Patients with moderate/severe exacerbation vs. patients without, HR (95% CI)** |
| Moderate/severe exacerbation Severe exacerbation Death up to Day 1,470 | 1.85 (1.70, 2.02)  1.95 (1.67, 2.28)  1.25 (1.06, 1.48) | 1.08 (1.00, 1.16)  1.02 (0.90, 1.15)  0.97 (0.83, 1.13) | 1.23 (1.14, 1.32)  1.44 (1.28, 1.63)  1.12 (0.97, 1.31) | 2.63 (2.44, 2.82)  2.18 (1.94, 2.46)  1.36 (1.18, 1.57) |

CI: confidence interval; CID: clinically important deterioration; FEV_1_: forced expiratory volume in 1 second; HR: hazard ratio; SGRQ: St. George’s Respiratory Questionnaire.

**Supplementary Table 4** Risk of exacerbation or death by confirmed clinically important deterioration status at Month 6 in the tiotropium and placebo arms, and at Month 12 in the tiotropium and placebo arms and total population

| **Outcome at Month 6** | **Patients with any CID event vs. patients without,  HR (95% CI)** | **Patients with FEV_1_ confirmed decline vs. patients without, HR (95% CI)** | **Patients with SGRQ confirmed deterioration vs. patients without, HR (95% CI)** | **Patients with moderate/severe exacerbation vs. patients without, HR (95% CI)** |
| --- | --- | --- | --- | --- |
| **Tiotropium**  Moderate/severe exacerbation Severe exacerbation Death up to Day 1,470 | 1.94 (1.76, 2.13)  1.79 (1.53, 2.09)  1.26 (1.03, 1.54) | 1.08 (0.92, 1.26)  0.94 (0.72, 1.23)  1.05 (0.76, 1.45) | 1.37 (1.19, 1.59)  1.74 (1.40, 2.16)  1.26 (0.93, 1.69) | 2.46 (2.22, 2.73)  1.96 (1.67, 2.30)  1.31 (1.06, 1.63) |
| **Placebo**  Moderate/severe exacerbation Severe exacerbation Death up to Day 1,470 | 1.64 (1.48, 1.80)  1.52 (1.30, 1.78)  1.15 (0.95, 1.39) | 1.08 (0.97, 1.21)  1.07 (0.89, 1.27)  1.08 (0.87, 1.34) | 1.21 (1.07, 1.38)  1.57 (1.30, 1.89)  1.27 (0.99, 1.62) | 2.24 (2.03, 2.48)  1.79 (1.54, 2.10)  1.13 (0.93, 1.38) |
| **Outcome at Month 12** | **Patients with any CID event vs. patients without,  HR (95% CI)** | **Patients with FEV_1_ decline vs. patients without,  HR (95% CI)** | **Patients with SGRQ deterioration vs. patients without, HR (95% CI)** | **Patients with moderate/severe exacerbation vs. patients without, HR (95% CI)** |
| **Overall population**  Moderate/severe exacerbation Severe exacerbation Death up to Day 1,470 | 2.02 (1.87, 2.18)  1.96 (1.72, 2.24)  1.31 (1.13, 1.53) | 1.12 (1.03, 1.21)  1.14 (0.99, 1.31)  1.07 (0.91, 1.27) | 1.27 (1.17, 1.39)  1.58 (1.38, 1.81)  1.38 (1.17, 1.64) | 2.63 (2.44, 2.82)  2.18 (1.94, 2.46)  1.36 (1.18, 1.57) |
| **Tiotropium**  Moderate/severe exacerbation Severe exacerbation Death up to Day 1,470 | 2.10 (1.89, 2.33)  2.10 (1.75, 2.52)  1.45 (1.17, 1.80) | 1.10 (0.96, 1.26)  1.17 (0.94, 1.47)  1.03 (0.77, 1.37) | 1.31 (1.15, 1.49)  1.64 (1.34, 2.01)  1.55 (1.21, 1.99) | 2.66 (2.40, 2.94)  2.26 (1.90, 2.67)  1.49 (1.21, 1.83) |
| **Placebo**  Moderate/severe exacerbation Severe exacerbation Death up to Day 1,470 | 1.91 (1.70, 2.14)  1.79 (1.47, 2.18)  1.16 (0.94, 1.43) | 1.08 (0.97, 1.21)  1.09 (0.91, 1.30)  1.06 (0.86, 1.32) | 1.22 (1.08, 1.37)  1.51 (1.26, 1.82)  1.25 (0.99, 1.57) | 2.58 (2.33, 2.86)  2.10 (1.77, 2.49)  1.24 (1.02, 1.51) |

CI: confidence interval; CID: clinically important deterioration; FEV_1_: forced expiratory volume in 1 second; HR: hazard ratio; SGRQ: St. George’s Respiratory Questionnaire.

**Supplementary Table 5** Risk of exacerbation or death by confirmed clinically important deterioration status calculated using clinically important deterioration event as a time-varying covariate

|  | **Patients with any CID event vs. patients without,  HR (95% CI)** | **Patients with confirmed FEV_1_ decline vs. patients without, HR (95% CI)** | **Patients with confirmed SGRQ deterioration vs. patients without, HR (95% CI)** | **Patients with moderate/severe exacerbation vs. patients without, HR (95% CI)** |
| --- | --- | --- | --- | --- |
| **Univariate Cox regression model** |  |  |  |  |
| Death up to Day 1,470 | 1.69 (1.42, 2.01) | 1.30 (1.13, 1.50) | 1.67 (1.45, 1.92) | 1.62 (1.40, 1.87) |
| **Stepwise Cox regression model*** |  |  |  |  |
| Death up to Day 1,470 | 1.59 (1.33, 1.90) | 1.62 (1.39, 1.88) | 1.84 (1.58, 2.13) | 1.39 (1.20, 1.61) |
| **Stepwise Cox regression model* including the three components** |  |  |  |  |
| Death up to Day 1,470 | N/A | 1.46 (1.26, 1.70) | 1.66 (1.43, 1.94) | 1.25 (1.08, 1.46) |

*Categorical variables included for selection in the stepwise Cox regression model were GOLD stage, LABA at baseline, ICS at baseline, LAMA at baseline, age, BMI, gender, smoking status and region; continuous variables were baseline SGRQ, baseline pre-bronchodilator FEV_1_, number of moderate/severe exacerbations in the previous year and severe exacerbations in the previous year.

BMI: body mass index; CI: confidence interval; CID: clinically important deterioration; FEV_1_: forced expiratory volume in 1 second; GOLD: Global Initiative for Chronic Obstructive Lung Disease; HR: hazard ratio; ICS: inhaled corticosteroid; LABA: long-acting β_2_-agonist; LAMA: long-acting muscarinic antagonist; SGRQ: St. George’s Respiratory Questionnaire.

**Supplementary Table 6** Patients with FEV_1_ decline or SGRQ deterioration in the total UPLIFT population 6, 12, 18, 24, 30, 36, and 42 months after the initial confirmed FEV_1_ decline or SGRQ deterioration: Tiotropium and placebo

|  | **Patients with confirmed FEV_1_ decline or SGRQ deterioration at time points after the initial decline  (available assessments)** | | | | | | | |
| --- | --- | --- | --- | --- | --- | --- | --- | --- |
|  | **Months after confirmed FEV_1_ decline or SGRQ increase** | | | | | | | |
|  | **6** | **12** | **18** | **24** | **30** | **36** | **42** | **48** |
| **FEV_1_ decline** |  |  |  |  |  |  |  |  |
| **Tiotropium** |  |  |  |  |  |  |  |  |
| No. of patients with decline, n (%) | 773/773 (100.0) | 473/659 (71.8) | 408/555 (73.5) | 327/457 (71.6) | 263/347 (75.8) | 195/261 (74.7) | 126/173 (72.8) | 85/110 (77.3) |
| Mean FEV_1_ change from baseline, L (SD) | -215 (100) | -182 (177) | -189 (193) | -197 (202) | -222 (209) | -230 (225) | -247 (231) | -227 (222) |
| Median FEV_1_ change from baseline (min, max) | -190 (-670, -40) | -170 (-1,000, 850) | -190 (-970, 630) | -200 (-1,070, 740) | -220 (-970, 470) | -220 (-1,050, 490) | -250 (-910, 380) | -220 (-870, 670) |
| **Placebo** |  |  |  |  |  |  |  |  |
| No. of patients with decline, n (%) | 1,151/1,151 (100.0) | 766/1,002 (76.4) | 683/892 (76.6) | 568/742 (76.5) | 491/619 (79.3) | 409/494 (82.8) | 293/376 (77.9) | 195/233 (83.7) |
| Mean FEV_1_ change from baseline, L (SD) | -228 (122) | -200 (176) | -210 (183) | -225 (191) | -245 (214) | -270 (223) | -264 (230) | -304 (221) |
| Median FEV_1_ change from baseline (min, max) | -190 (-1,090, -100) | -190 (-1,240, 650) | -200 (-1,000, 500) | -210 (-920, 430) | -240 (-1,190, 650) | -260 (-1,200, 450) | -250 (-980, 460) | -300 (-920, 320) |
| **SGRQ deterioration** |  |  |  |  |  |  |  |  |
| **Tiotropium** |  |  |  |  |  |  |  |  |
| No. of patients with deterioration, n (%) | 812/812 (100.0) | 527/691 (76.3) | 441/600 (73.5) | 360/497 (72.4) | 278/381 (73.0) | 199/273 (72.9) | 119/160 (74.4) |  |
| Mean SGRQ change from baseline (SD) | 12.2 (7.2) | 10.6 (10.5) | 10.5 (11.5) | 11.1 (12.4) | 10.8 (12.2) | 11.6 (12.7) | 12.1 (13.1) |  |
| Median SGRQ score change from baseline (min, max) | 10.1 (4.0, 44.8) | 9.3 (-25.5, 50.8) | 9.2 (-38.4, 67.8) | 10.3 (-33.6, 69.6) | 10.3 (-36.6, 54.6) | 10.9 (-33.8, 48.2) | 11.8 (-22.1, 41.7) |  |
| **Placebo** |  |  |  |  |  |  |  |  |
| No. of patients with deterioration, n (%) | 973/973 (100.0) | 613/819 (74.8) | 500/701 (71.3) | 416/570 (73.0) | 351/462 (76.0) | 263/344 (76.5) | 184/228 (80.7) |  |
| Mean SGRQ change from baseline (SD) | 12.7 (7.7) | 10.7 (10.8) | 9.9 (11.4) | 10.4 (12.3) | 11.7 (12.9) | 11.9 (12.9) | 12.4 (12.6) |  |
| Median SGRQ score change from baseline (min, max) | 10.6 (4.0, 53.1) | 9.7 (-48.4, 53.9) | 9.4 (-44.2, 48.8) | 9.9 (-44.5, 66.0) | 11.3 (-35.7, 53.6) | 10.9 (-30.1, 50.1) | 12.0 (-29.8, 47.3) |  |

FEV_1_: forced expiratory volume in 1 second; SD: standard deviation; SGRQ: St. George’s Respiratory Questionnaire.
For patients with confirmed decline at Month 1, their assessments at Months 6, 12, 18, 24, 30, 36, 42 and 48 are used as time from first confirmed deterioration. Unscheduled visits were excluded for this analysis. Only patients with confirmed FEV_1_ decline or confirmed SGRQ deterioration and their available assessments at each time point after the deterioration were included in the analysis.

**Supplementary Table 7** Timing of FEV_1_ decline and SGRQ deterioration relative to each other in tiotropium and placebo arms

|  | **Tiotropium** | **Placebo** |
| --- | --- | --- |
| Patients with both confirmed FEV_1_ decline and confirmed SGRQ deterioration, n (%)  On same assessment  FEV_1_ decline before SGRQ deterioration  SGRQ deterioration before FEV_1_ decline  Moderate/severe exacerbation before FEV_1_ and SGRQ deterioration | 516 (100.0) 92 (17.8) 199 (38.6) 225 (43.6) 222 (43.0) | 828 (100.0) 148 (17.9) 421 (50.8) 259 (31.3) 324 (39.1) |
| Patients with confirmed FEV_1_ decline and no confirmed SGRQ deterioration, n (%)  Moderate/severe exacerbation before FEV_1_ decline  Unconfirmed SGRQ deterioration  On same assessment as confirmed FEV_1_ decline  Before confirmed FEV_1_ decline | 514 (100.0) 225 (43.8) 133 (25.9) 12 (2.3) 61 (11.9) | 646 (100.0) 235 (36.4) 169 (26.2) 22 (3.4) 55 (8.5) |
| Patients with confirmed SGRQ and no confirmed FEV_1_ deterioration, n (%)  Moderate/severe exacerbation before SGRQ deterioration  Unconfirmed FEV_1_ deterioration  On same assessment as confirmed SGRQ deterioration  Before confirmed SGRQ deterioration | 561 (100.0) 321 (57.2) 126 (22.5) 21 (3.7) 65 (11.6) | 434 (100.0) 253 (58.3) 158 (36.4) 29 (6.7) 86 (19.8) |

FEV_1_: forced expiratory volume in 1 second; SGRQ: St. George’s Respiratory Questionnaire.

**Supplementary Figure 1** Time to first event for (A) trough FEV_1_ decline ≥100 mL, (B) SGRQ increase ≥4 units and (C) moderate/severe exacerbation

**(A)**

**(B)**

**(C)**

CI: confidence interval; FEV_1_: forced expiratory volume in 1 second; SGRQ: St. George’s Respiratory Questionnaire.

References

1. Global Initiative for Chronic Obstructive Lung D: **Global Strategy for the Diagnosis, Management, and Prevention of Chronic Obstructive Pulmonary Disease: 2018 Report.** vol. 2018.
